# A rare double ALK fusion variant EML4-ALK and CDK15-ALK in lung adenocarcinoma and response to crizotinib: A case report

**DOI:** 10.1097/MD.0000000000022631

**Published:** 2020-11-06

**Authors:** Jun Guo, Junping Shi, Ming Yao, Yi Jin, Dengxiang Liu, Weiling Liu, Kai Wang, Da Jiang

**Affiliations:** a Department of Internal Medicine-Oncology, Xingtai People's Hospital, Xingtai, Hebei Province, China; b OrigiMed Co. Ltd, Shanghai, China; c Department of Oncology, Xingtai People's Hospital, Xingtai, Hebei Province; d Department of Medical Oncology, the Fourth Hospital of Hebei Medical University, Shijiazhuang, Hebei Province, China.s.

**Keywords:** EML4-ALK, CDK15-ALK, double fusions, lung adenocarcinoma, crizotinib

## Abstract

**Rationale:**

The anaplastic lymphoma kinase (ALK) fusion has been identified to be a driver gene in lung cancer, and serves as important diagnostic and therapeutic targets. Owing to the advanced sequencing technologies, new partner genes of ALK have been constantly detected.

**Patient concerns:**

A 55-year-old Chinese woman went to our hospital because of cough and expectoration for 1 year. The patient had no fever, chest pain and hemoptysis.

**Diagnoses:**

She was diagnosed with lung adenocarcinoma. Because she had no operational condition, combination chemotherapy with docetaxel and cisplatin (CP) for 4 cycles was adopted. However, computed tomography (CT) scan indicated progression disease (PD). To explore possibility of targeted therapy, the tumor samples were subjected to next-generation sequencing (NGS), and a rare double ALK fusion variant EML4-ALK and CDK15-ALK was identified.

**Interventions and outcomes:**

The patient subsequently received crizotinib treatment, and achieved partial response (PR). No significant drug related adverse reactions were found during crizotinib treatment. The progression-free survival achieved 23 months.

**Lessons:**

Together, we identified a rare double ALK fusion variant, EML4-ALK and CDK15-ALK, in a patient with lung adenocarcinoma. The patient benefited from crizotinib treatment, which could provide a certain reference for the patients with such gene alteration.

## 1 Introduction

Lung cancer is a malignant disease with high morbidity and mortality. Lung adenocarcinoma originates from the epithelium and belongs to non-small cell lung cancer (NSCLC).^[1]^ In the past decade, with the development of next generation sequencing (NGS) technology, various cancer driven genes have been identified. Anaplastic lymphoma kinase (ALK) fusions are detected in many cancers with the highest detection rate in NSCLC.^[2]^ ALK fusion is an important driver gene in NSCLC, fusion partners of which have been discovered gradually, including EML4, CMTR1, KLC1, TNIP2 and CUX1.^[3–7]^ However, ALK double fusion in NSCLC is still rare even though ALK fusion is detected in about 4% of lung adenocarcinoma. Here we report a rare double ALK fusion variant, EML4-ALK and CDK15-ALK in a patient with lung adenocarcinoma who responded well to crizotinib.

## 2 Case report

**Figure 1:**
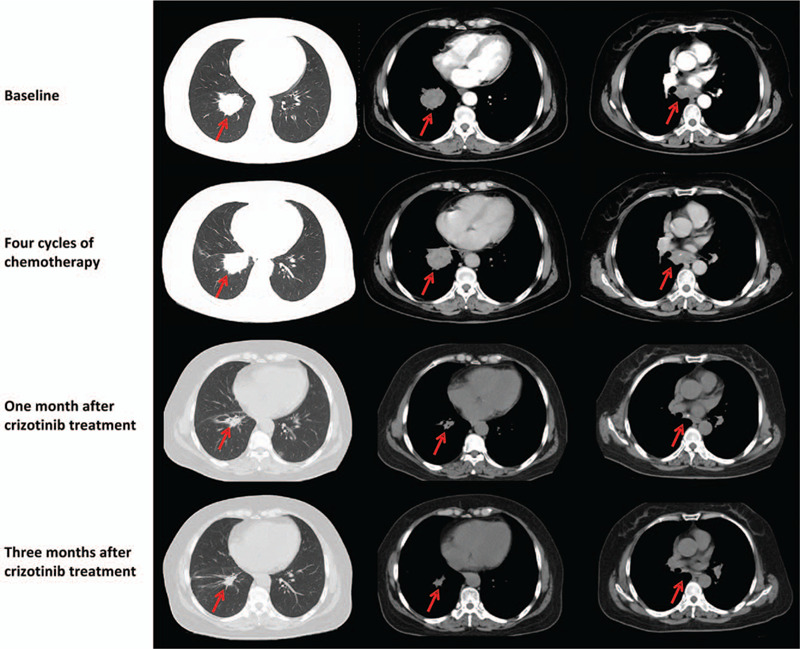
Computed tomography (CT) scans of patient before and after crizotinib treatment.

The patient was a 55-year-old female with no smoking history and no family history of cancer. In February 2017, she went to our hospital because of cough and expectoration for 1 year. The patient had no fever, chest pain and hemoptysis. Chest enhanced CT showed a mass lesion in the upper lobe of the right lung and multiple enlarged lymph nodes in the mediastinum. The pathological diagnosis of the biopsy was low differentiated adenocarcinoma, stage IIIB. Because the patient had no operational condition, combination chemotherapy with docetaxel and cisplatin (CP) for 4 cycles was adopted. CT re-examination showed that the tumor was enlarged and the assessment was progression disease (PD) according to RECIST 1.1 (Fig. 1).

**Figure 2:**
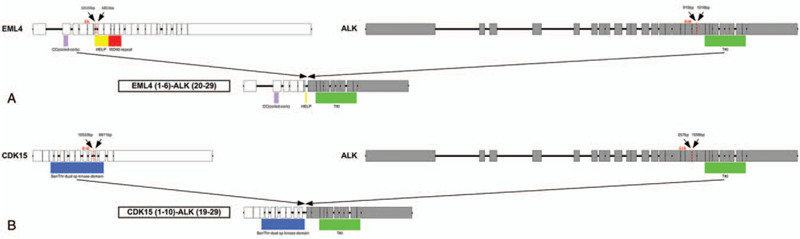
Schematic diagram of the double fusions. (A) EML4 and ALK genomic DNA structures, breakpoint location and fusion gene structure. (B) CDK15 and ALK genomic DNA structures, breakpoint location and fusion gene structure.

To explore possibility of targeted therapy, the tumor samples were subjected to genomic sequencing by using next-generation sequencing (NGS), and the results showed that this patient carried two ALK fusions simultaneously, EML4-ALK (V3) and CDK15-ALK (Fig. 2). EML4-ALK fusion was generated by the fusion of exons 1-6 of EML4 to exons 20-29 of ALK while CDK15-ALK fusion was generated by the fusion of exons 1-10 of CDK15 to exons 19-29 of ALK. The function predication showed that the fusions had complete ALK kinase domain which may lead to the activation of ALK kinase. In August 2017, the patient subsequently received crizotinib (250 mg, bid po) treatment. After one month, her cough and expectoration were alleviated a lot. CT re-examination showed the size of tumor was reduced and the assessment was partial response (PR) according to RECIST 1.1 (Fig. 1). The patient continued to take crizotinib, and after 3 months, the tumor continued to shrink, and no new lesions were found (Fig. 1). As of July, 2019, the patient was still taking crizotinib and no significant drug related adverse reactions were found. The progression-free survival achieved 23 months. After that, the patient did not come to our department for re-examination and was lost to follow-up. Written informed consent for the study was obtained from the patient.

## 3 Discussion

In this report, we identified a rare double ALK fusion, EML4-ALK and CDK15-ALK, in the patient with lung adenocarcinoma. CDK15-ALK fusion was a recently identified fusion. CDK15 gene encodes cyclin dependent kinase, and involves in the resistance to tumor necrosis factor induced apoptosis.^[8]^ EML4-ALK fusion is the classic and most common fusion mutation in NSCLC. In 2007, SODA et al. found the EML4-ALK fusion gene for the first time in NSCLC, which could lead to the occurrence of cancer. Thus, the ALK fusion has been considered as a very important lung cancer driver gene.^[9]^

ALK inhibitors are widely used in cancer-targeted therapy now. Crizotinib is a small-molecule tyrosine kinase inhibitor (TKI) which actively inhibits ALK, MET, and ROS1. Crizotinib showed significant activity in ALK-driven tumors and was FDA approved for ALK-positive advanced NSCLC.^[10]^ However, different ALK fusion variants have different responses to crizotinib. Based on the different cleavage sites, EML4-ALK fusion gene variants can be divided into several subtypes, V1, V2, V3a/b/c, V4, V5a/b, V6a/b, V7, V8a/b/d and V10. Among them, subtypes of V1 (32-33.3%), V2 (8-11.1%) and V3a/b (32-44%) are the most common mutation subtypes, while other EML4-ALK fusion variants only account for 5.6% to 12%.^[11,12]^

Several studies have explored the potential association of EML4-ALK fusion with therapeutic response, but the results are inconclusive. Lei et al did not observe any significant difference in the efficacy of crizotinib between patients with the EML4-ALK fusion variant V3, V1 and other less frequent variants V2.^[13]^ Yoshida et al found that the objective response rate (ORR) and disease control rate (DCR) of the EML4-ALK variant V1 responding to Crizotinib were 74% and 95% respectively, while other ALK fusions were 63% and 63%.^[11]^ Another clinical study showed that the 2-year PFS of NSCLC patients with V3a/b was 26.4%, significantly lower than that other subgroups. It suggested that EML4-ALK variants 3a/b may be a major source of ALK inhibitor resistance in the clinic.^[12]^ In addition, in vitro experiments, the protein encoded by EML4-ALK V3 fusion showed longer half-life and stronger carcinogenic signals than other mutant subtypes, and this may be another reason for the worse prognosis of the V3 patients, which is not related to the drug resistance.^[14]^ Christopoulos et al demonstrated that EML4-ALK V3 fusion is a high-risk feature for ALK^+^ NSCLC.^[15]^ In this study, the patient carried EML4-ALK V3 mutation and CDK15-ALK fusion, but she still showed a good response to crizotinib treatment. This is a very lucky thing, but how long this response last needs further observation. Thus, continuous studies on the form of ALK fusion, the correlation between them and the sensitivity of targeted drugs have great clinical significance. Moreover, with accumulation of related information, it is necessary to include molecular testing beyond FISH in the future diagnostic guidelines for ALK^+^ NSCLC and to develop more effective strategies for management of higher-risk, V3-positive cases.

In summary, we represented a rare double ALK fusion composed of a classic fusion variant of EML4-ALK and a newly discovered CDK15-ALK in a lung adenocarcinoma. This report fully demonstrated the complexity and diversity of ALK fusion. Also, the curative effect of crizotinib in the treatment of the patient provided a certain therapeutic reference for the patients with such gene alterations.

## Author contributions

**Conceptualization:** Jun Guo, Da Jiang

**Data curation:** Junping Shi, Ming Yao, Yi Jin

**Investigation:** Dengxiang Liu, Weiling Liu, Kai Wang

**Methodology:** Jun Guo, Junping Shi, Ming Yao

**Validation:** Yi Jin, Dengxiang Liu

**Writing – original draft:** Jun Guo, Da Jiang

**Writing – review & editing:** Jun Guo, Junping Shi, Ming Yao, Yi Jin, Dengxiang Liu, Weiling Liu, Kai Wang, Da Jiang
